# Simplified ^89^Zr-Labeling Protocol of Oxine (8-Hydroxyquinoline) Enabling Prolonged Tracking of Liposome-Based Nanomedicines and Cells

**DOI:** 10.3390/pharmaceutics13071097

**Published:** 2021-07-18

**Authors:** Andras Polyak, Jens P. Bankstahl, Karen F. W. Besecke, Constantin Hozsa, Wiebke Triebert, Rajeswara Rao Pannem, Felix Manstein, Thomas Borcholte, Marcus Furch, Robert Zweigerdt, Robert K. Gieseler, Frank M. Bengel, Tobias L. Ross

**Affiliations:** 1 Department of Nuclear Medicine, Hannover Medical School, 30625 Hannover, Germany; bankstahl.jens@mh-hannover.de (J.P.B.); Bengel.Frank@mh-hannover.de (F.M.B.); ross.tobias@mh-hannover.de (T.L.R.); 2 Rodos Biotarget GmbH, Medical Park Hannover, 30625 Hannover, Germany; k.besecke@solmic-biotech.de (K.F.W.B.); constantin.hozsa@freenet.de (C.H.); rajeswararao.pannem@biolife-group.com (R.R.P.); thomas@borcholte.de (T.B.); marcus.furch@biolife-group.com (M.F.); rk.gieseler@gmx.de (R.K.G.); 3 SolMic BioTech GmbH, 40225 Düsseldorf, Germany; 4 Leibniz Research Laboratories for Biotechnology and Artificial Organs (LEBAO), 30625 Hannover, Germany; triebert.wiebke@mh-hannover.de (W.T.); manstein.felix@mh-hannover.de (F.M.); zweigerdt.robert@mh-hannover.de (R.Z.); 5 Department of Cardiac, Thoracic, Transplantation and Vascular Surgery (HTTG), Hannover Medical School, 30625 Hannover, Germany; 6 Bioloving GmbH & Co KG, 69126 Heidelberg, Germany; 7 Department of Internal Medicine, and Laboratory of Immunology & Molecular Biology, University Hospital, Knappschaftskrankenhaus, Ruhr University Bochum, 44801 Bochum, Germany

**Keywords:** nanoparticles, liposomes, cell labeling, imaging, PET, nanomedicine, human induced pluripotent stem cells (hiPSCs)

## Abstract

In this work, a method for the preparation of the highly lipophilic labeling synthon [^89^Zr]Zr(oxinate)_4_ was optimized for the radiolabeling of liposomes and human induced pluripotent stem cells (hiPSCs). The aim was to establish a robust and reliable labeling protocol for enabling up to one week positron emission tomography (PET) tracing of lipid-based nanomedicines and transplanted or injected cells, respectively. [^89^Zr]Zr(oxinate)_4_ was prepared from oxine (8-hydroxyquinoline) and [^89^Zr]Zr(OH)_2_(C_2_O_4_). Earlier introduced liquid–liquid extraction methods were simplified by the optimization of buffering, pH, temperature and reaction times. For quality control, thin-layer chromatography (TLC), size-exclusion chromatography (SEC) and centrifugation were employed. Subsequently, the ^89^Zr-complex was incorporated into liposome formulations. PET/CT imaging of ^89^Zr-labeled liposomes was performed in healthy mice. Cell labeling was accomplished in PBS using suspensions of 3 × 10^6^ hiPSCs, each. [^89^Zr]Zr(oxinate)_4_ was synthesized in very high radiochemical yields of 98.7% (96.8% ± 2.8%). Similarly, high internalization rates (≥90%) of [^89^Zr]Zr(oxinate)_4_ into liposomes were obtained over an 18 h incubation period. MicroPET and biodistribution studies confirmed the labeled nanocarriers’ in vivo stability. Human iPSCs incorporated the labeling agent within 30 min with ~50% efficiency. Prolonged PET imaging is an ideal tool in the development of lipid-based nanocarriers for drug delivery and cell therapies. To this end, a reliable and reproducible ^89^Zr radiolabeling method was developed and tested successfully in a model liposome system and in hiPSCs alike.

## 1. Introduction

The development of nuclear-imaging-guided, liposome-based nanomedicines or nanotheranostics occasionally requires prolonged tracing time due to the slower kinetics, enhanced circulation and excretion time of the candidate compounds. Zirconium-89 (^89^Zr)-labeled oxine (8-hydroxyquinoline) has recently emerged as a favorable positron emission tomography (PET) alternative to single photon emission computed tomography (SPECT) indium-111-labeled oxine [1]. The positron emitter ^89^Zr (T_½_ = 78.4 h) offers the opportunity of tracking cells or lipid-based nanomedicines by PET for up to one week. 

Oxinates of the transition radiometals gallium-68, zirconium-89 and indium-111 ([^68^Ga]Ga(oxinate)_3_, [^89^Zr]Zr(oxinate)_4_, [^111^In]In(oxinate)_3_) are highly lipophilic molecules that can be incorporated into lipid-bilayered nanovesicles (liposomes) and living cells under neutral conditions. Using this mechanism, more recent studies disseminated [^89^Zr]Zr(oxinate)_4_-related cell tracing by applying various labeling protocols from 60% to 97% labeling efficiency of the prelabeled oxinate [2,3,4,5,6] and complex liposome labeling by combining this method with a liposome-incorporated bifunctional chelator [7].

Based upon these findings, the present study focused on simplifying and optimizing the [^89^Zr]Zr(oxinate)_4_ production. Liquid–liquid extraction or solvent extraction methods were applied in which the ^89^Zr radioisotope was transferred from the aqueous raffinate to the chloroform solution of chelating 8-hydroxyquinoline. We tested different buffer environments, temperatures, reaction times and stirring methods. The present study was primarily aimed at identifying the key factors to allow for a reproducible and robust synthesis protocol at the highest isotope incorporation yield, while minimizing time and effort. The validity of the established method was verified by PET on a model liposome system and via labeling trials of human induced pluripotent stem cells (hiPSCs). As of more recently, human iPSCs and their progenies can be generated with high efficiency by advanced protocols [8,9] and have great potential for regenerative medicine. However, progress towards the clinical translation of hiPSCs requires efficient labeling technologies to monitor aspects of transplantation safety and efficiency [10]. The established protocols were employed in advanced nanomedicinal and stem cell transplantation studies to be presented elsewhere.

## 2. Materials and Methods

### 2.1. Oxine Labeling

^89^Zr was produced at the BV Cyclotron VU (Vrije Universiteit Amsterdam, Amsterdam, The Netherlands) as [^89^Zr]Zr(OH)_2_(C_2_O_4_) (oxalate) in 1 M oxalic acid that was diluted with 500–1000 µL aq. dest., neutralized with 1 M NaOH and buffered with pH 7.4 phosphate-buffered saline (PBS) and pH 7.5 0.5 M 4-(2-hydroxyethyl)-1-piperazineethanesulfonic acid (HEPES). The samples’ radioactivity was measured in a Veenstra dose calibrator. Silica gel impregnated glass fiber (ITLC-SG) chromatography plates were purchased from Merck. [^89^Zr]Zr(oxinate)_4_ at an activity of 5–40 MBq was prepared from 500–1500 µL of 2–6 mg/mL oxine (8-hydroxyquinoline) solution in chloroform by the liquid–liquid (solvent) extraction method. Reactions were performed at RT, 50 °C or 60 °C, respectively. The [^89^Zr]Zr(oxinate)_4_-labeling yield was calculated from the measured activities of the separated aqueous raffinates and chloroform extracts after different times (5, 10, 15, 20, 30, 60 min, 2 h and24 h) of extensive mixing in glass reaction vessels. After extraction, [^89^Zr]Zr(oxinate)_4_ was crystallized by evaporation of chloroform at 60 °C under a N_2_ stream and was redissolved in ethanol and dimethyl sulfoxide (DMSO).

### 2.2. Liposome Formulation and Labeling

The model liposome for the radiolabeling studies was TargoSphere^®^ [11,12], an umbrella term coined for various lipid-based nanocarriers developed by Rodos Biotarget. The thin-film hydration method followed by extrusion [13] was used for preparing liposomes. In brief, phospholipids were dissolved, the stock solutions were combined in round-bottomed flasks, and lipid films were subsequently formulated by removing the solvents by means of a rotary evaporator. The resulting dry films were hydrated with PBS, and the crude samples were extruded through polycarbonate membranes with a pore size of 200 nm (Whatman^®^ Nucleopore™ Track-Etched Membrane), followed by extrusion through 50 nm. For radiolabeling, 10–100 µL liposomal aliquots of 30–35 µg/µL lipid concentration were added to [^89^Zr]Zr(oxinate)_4_.

### 2.3. Characterization of Liposomes

Particle size distributions were determined by dynamic light scattering (DLS) using a Malvern Zetasizer Nano ZS device (Malvern Panalytical), thus gaining the polydispersity index (PDI) and ζ potential. The labeling yield of the [^89^Zr]Zr(oxinate)_4_–liposome complex was checked after 5, 10, 15, 20, 30 and 60 min as well as after 6, 18 and 24 h; the reactions were performed at RT or 60 °C, respectively. To this end, we employed thin-layer chromatography (TLC), size-exclusion chromatography (SEC) and separation by centrifugation. As a thin layer, ITLC-SG strips (Merck, Darmstadt, Germany) were developed by 0.1 M citrate buffer, chloroform, chloroform–MeOH 5% and 20 mmol EDTA solutions [14]. Reference samples were original [^89^Zr]Zr(OH)_2_(C_2_O_4_) diluted by PBS and [^89^Zr]Zr(oxinate)_4_ solutions. SEC was performed using a PD-10 MidiTrap G-25 column (GE Healthcare Life Sciences, Braunschweig, Germany).

### 2.4. PET Imaging of Liposomes

PET imaging was used to evaluate the in vivo integrity of the [^89^Zr]Zr(oxinate)_4_–liposome complex. Labeled liposomes were injected intravenously (IV) in healthy C57BL/6 mice (*n* = 5). As a control, PBS-buffered [^89^Zr]Zr(OH)_2_(C_2_O_4_) was applied in two animals. Injected activities and volumes were 5.0 MBq in 100 µL per animal. The final lipid concentration of the solutions applied in vivo was 0.91–0.94 µg/µL. Mice were then subjected to serial PET imaging using a small animal microPET/CT system (Inveon DPET and CT120; Siemens Healthineers, Erlangen, Germany). Right after IV application, dynamic PET images were acquired over a 60 min period, and a static PET acquisition was performed 24 h later. Values of the I.D./g tissue (i.e., injected activity per gram unit of tissue) were determined from the region of interest per volume of interest (ROI/VOI) selections from spatial PET images using PMOD software (PMOD Technologies, Zürich, Switzerland).

### 2.5. Cell Culture and Labeling

The hiPSC lines hHSC_1285i_iPS2 (MHHi006-A [15]; or MHHi001-A-5 [16]) were cultured by conventional surface-adherent 2D culture and in 3D suspension culture, as previously described [9]. In brief, cryopreserved hiPSCs were thawed and cultured over 2–3 passages on Geltrex^®^-coated T-flasks in Essential 8 medium (E8) with Rho-kinase inhibitor (RI). Subsequently, hiPSCs were dissociated using Accutase^TM^ treatment; ~10 million single cells were inoculated in 20 mL E8 + RI in a 125 mL Erlenmeyer flask and placed on a horizontal shaker rotating at 70 rpm, placed in a conventional incubator at 37 °C, 5% CO_2_ and 95% RH for 2–3 days to allow hiPSC aggregation and expansion in suspension. The prelabeled [^89^Zr]Zr(oxinate)_4_ was redissolved in DMSO and 30, 60 or 90 µL of this solution was added to 3 mL aliquots of ~3 × 10^6^ cells in suspension (in individual wells in a 6-well plate), reaching DMSO concentrations of 1%, 2% or 3%, respectively. Cells were incubated for 5, 10, 15, 30 and 60 min as well as 6 and 24 h while applying stirring at 100 rpm. Cells were collected by pelleting at 100× *g* for 5 min and resuspended in PBS. Labeling yields were determined by assessing both percentages of radioactivity remaining in the supernatant and cell-bound radioactivity.

## 3. Results

### 3.1. [^89^Zr]Zr(oxinate)_4_ Labeling

**Figure 1:**
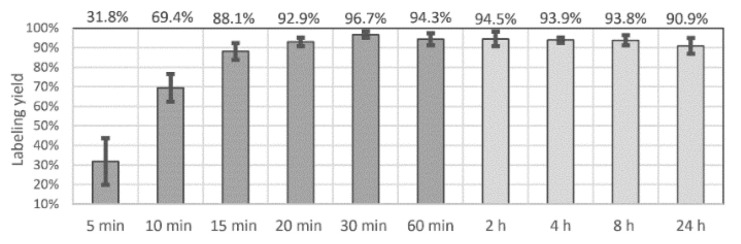
Radiolabeling yields of [^89^Zr]Zr(oxinate)_4_ up to extraction at 60 min, and in the subsequent stability samplings in PBS (2 to 24 h).

From the different pH adjustment methods, the 0.5 M HEPES (pH 7.5)-buffered [^89^Zr]Zr(oxinate)_4_ synthesis rendered the maximum radiochemical yield of 98.7%, which was achieved after 30 min of high-speed stirring (Figure 1; Table S1). Optimizing the volume ratios and mixing circumstances, the average radiochemical yield was 96.7% (± SD 1.6%) for attempts over 30 min, while the earlier samplings showed average radiochemical yields of 88.1% ± SD 4.3% and 92.9% ± SD 2.2% after 15 min and 20 min, respectively (max. 95.9%). The highest distribution ratio resulted when applying a 1.0:2.4 (*v*/*v*) chloroform–water phase ratio. In this reaction mixture, the oxine concentration was set to 3 mg/mL in 500 µL chloroform, and the aqueous component (~1200 µL) was obtained by diluting 5–40 µL of the original 1 M oxalic acid [^89^Zr]Zr(OH)_2_(C_2_O_4_) solution with 500 µL water, neutralized by 16 µL of 1 M NaOH, and buffered with 700 µL 0.5M HEPES (pH 7.5). The turbulent dispersion was maintained for a maximum of 60 min (avg. 94.3%, ± SD 3.1%); then, the raffinate was manually separated from the extract, and the two sample activities were measured by dose calibration. The chloroform exacts were evaporated for 15 min at RT under a N_2_ stream. For 24 h radiochemical stability evaluations, [^89^Zr]Zr(oxinate)_4_ was redissolved with 30 µL DMSO post-extraction and intermittently diluted with PBS to 2 mL. Average yields proved to be 94.5% (±SD 3.7%), 93.0% (±SD 1.4%), 93.8% (±SD 2.5%) and 90.9% (±SD 4.0%) after 2, 4, 8 and 24 h of labeling, respectively (Figure 1). Highest yields were obtained when using asymmetrically shaped vials and applying maximal magnetic stirring at 1500 rpm.

Of note, the same raffinate solvent settings at 1:1 (*v*/*v*) and 1.5:1.0 (*v*/*v*) chloroform–water ratios resulted in remarkably lower yields and higher deviations (avg. 60.7% ± SD 20.1% at 1:1 ratio and avg. 45.7% ± SD 21.5% at 1.5:1.0 ratio). Attempts with a 1:1 (*v*/*v*) chloroform–water ratio using PBS-buffer or buffer-free (NaOH-adjusted) raffinates also resulted in poor yields after a mixing time of 30 min (avg. 32.5% ± SD 18.6% and 46.8% ± SD 26.9%, respectively).

### 3.2. Characterization of Liposomes

The mean diameter of the TargoSphere^®^ formulation employed proved to be 91.51 nm (Z-average: 89.12 nm), with a low 0.209 PDI. Measured ζ potential value was −0.0612 ± SD 0.0950. For a particle size distribution histogram, see Figure S3.

### 3.3. Liposome Labeling

Radioanalytics of unchelated ^89^Zr, [^89^Zr]Zr(oxinate)_4_ and [^89^Zr]Zr(oxinate)_4_ liposomes proved to be most effective and most selective when ITLC-SG strips were developed by 0.1 M citrate buffer and chloroform. In this setup, the [^89^Zr]Zr(oxinate)_4_–liposome complex remained at the origin (*R_f_* = 0) in citrate buffer, and non-colloidal components migrated with the solvent front (SF) (*R_f_* ≈ 1). Chloroform used as the mobile phase could separate the unchelated ^89^Zr (*R_f_* = 0) from the [^89^Zr]Zr(oxinate)_4_ (*R_f_* ≈ 1) and the [^89^Zr]Zr(oxinate)_4_ liposomes (*R_f_* ≈ 1). The model liposome system slowly incorporated the [^89^Zr]Zr(oxinate)_4_. Over 30 and 60 min, maximum incorporation yields were 44.2% ± SD 7.7% or 59.2% ± SD 9.2%, respectively. PET studies were performed at the highest internalization yields (i.e., 98.1% ± SD 1.8%) obtained upon 18 h incubation.

### 3.4. MicroPET Studies of Liposomes

**Figure 2:**
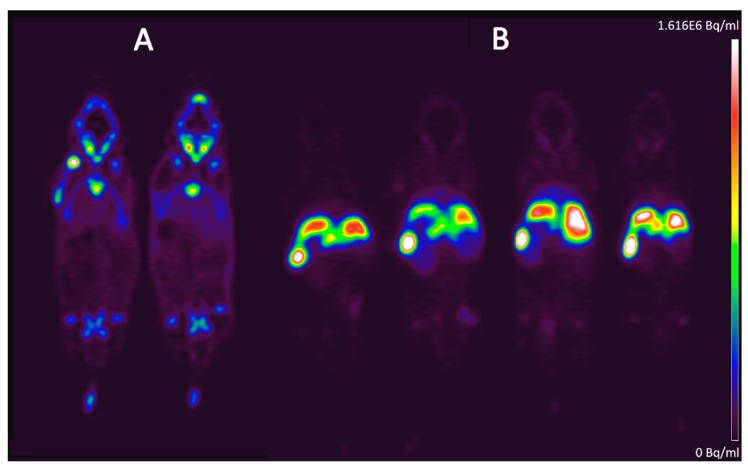

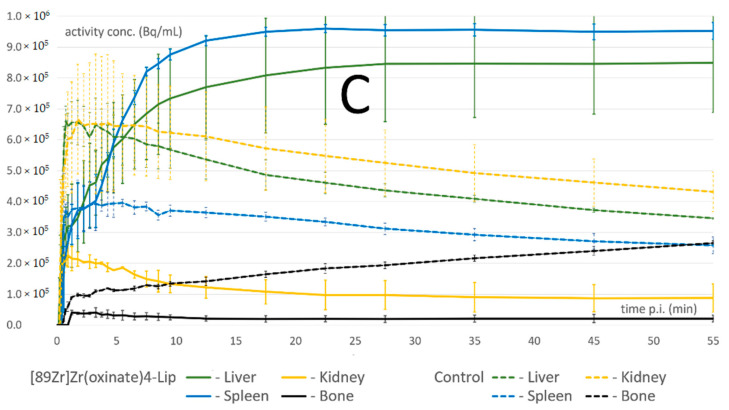
(**A**) Representative PET slices of two mice from the [^89^Zr]Zr(OH)_2_(C_2_O_4_) control group 24 h post-injection, and (**B**) of four mice that received the [^89^Zr]Zr(oxinate)_4_–liposomes. (**C**) Time–-activity curves of selected organs (liver, spleen, kidney and bone) generated from the dynamic PET imaging data over 55 min post-injection.

PET studies confirmed the radiochemical stability and colloidal integrity of the labeled complex after IV administration. Most of the injected radioactivity quickly accumulated in organs of the reticuloendothelial system within the first 15 min, while only moderate radioactivity was detected in other organs (Figure 2). The fast kinetics and early biodistribution of the [^89^Zr]Zr(oxinate)_4_ liposomes compared to the [^89^Zr]Zr(OH)_2_(C_2_O_4_) over 60 min p.i. are depicted in the time–activity curves generated from the dynamic PET imaging data (Figure 2C). Different uptake kinetics and organ distributions are clearly visible, predominately demonstrating hepatic, splenic and renal uptake. Specifically, high uptake by liver and spleen was observed over the entire 24 h tracing period.

At 24 h scans, 27.07% I.D./g tissue (±SD 2.64%) was found in the liver and 70.94% I.D./g tissue (±SD 11.33%) in the spleen. In the [^89^Zr]Zr(OH)_2_(C_2_O_4_)-injected control group, 1.22% I.D./g liver (±SD 0.11%) and 1.40% I.D./g spleen (±SD 0.02%) were detected (Figure 2A,B). Since the liver’s Kupffer cells comprise 80–90% of all macrophages, thus constituting the body’s largest macrophage population [17,18], the liver’s macrophage pool is thus presumed to be less saturated than the splenic macrophage subsets.

This pharmacokinetic profile and the 24 h organ distribution characteristics unequivocally corresponded to a nanosized labeled compound that was slowly released from the hepatic and splenic macrophages and excreted renally: the 24 h kidney uptake was 8.77% I.D./g (±SD 10.75%) compared to the control group with 1.24% I.D./g (±SD 0.01%). The 6.80% I.D./g (±SD 3.07%) lung activity indicated a low ratio of an aggregated particle fraction compared to the control group with 1.36% I.D./g (±SD 0.03%) corresponding values [18]. PET images of the [^89^Zr]Zr(OH)_2_(C_2_O_4_) control group showed skeletal uptake by the spine, extremities and skull.

### 3.5. Cell Labeling

HiPSCs quickly internalized the prelabeled lipophilic agent. The yield was 22.0% ± 1.8% at 5 min and 42.8% ± 3.8% at 10 min in 3% DMSO samplings, and the bound activity saturated at 15 and 30 min (52.5% ± 2.0% vs. 53.3% ± 2.2%, respectively). The yield was 49.9% ± 4.2 % at 60 min, while longer incubations resulted in lower yields (6 h: 40.5% ± 2.1%; 24 h: 39.0% ± 2.8%). Samples with lower DMSO and prelabeled agent concentrations followed similar internalization kinetics at slightly different maximum yields. The maximal bound ratio was 50.9% ± 4.0% and 47.6% ± 0.6% at 15 and 30 min in the 1% DMSO samples and 53.3% ± 2.8% and 50.6% ± 2.5% at 15 and 30 min in the 2% DMSO samples.

## 4. Discussion

More recent studies disseminated [^89^Zr]Zr(oxinate)_4_ radiolabeling by applying variable labeling protocols. For cell labeling, Charoenphun et al. first presented a protocol that employed a different buffering method, which resulted in ~60% labeling efficiency of the prelabeled oxinate [2]. Sato and colleagues published a more complex process that obtained ^89^ZrCl_4_ from ^89^Zr-oxalate, including an ion exchange step prior to solvent extraction [3]. Weist et al. reached an 82–92% labeling yield [4], while Patrick and colleagues achieved around 74% efficiency [5] using variable buffer and extraction settings. Man et al. reached [^89^Zr]Zr(oxinate)_4_-labeling yields similar to those achieved in the present study by applying the formulation of a more complex kit prior to cell labeling studies [6]. For liposome tracking, Li et al. [7] combined oxine prelabeling with a second liposome-incorporated bifunctional chelator deferoxamine [19], and then introduced a post-purification step for obtaining the final product. In contrast to all these approaches, here we provide a more simple, reproducible [^89^Zr]Zr(oxinate)_4_-labeling protocol for prospective liposome and cell-imaging investigations that requires no further post-purification process.

Besides the importance of appropriate buffering, our study illustrated the importance of proper mixing during the solvent extraction. Specifically, maximum ^89^Zr chelation requires a maximum distribution ratio, which cannot be reached by laminar mixing. Turbulent vortexing must be maintained, thus maximizing the liquid–liquid interface, preferably not in a “v-shape” microreaction vessel, but in an asymmetrically shaped liquid container at high-speed vortexing. For comparison, non-turbulent mixing resulted in either low or highly variable labeling yields. By considering these crucial process parameters, we obtained a reliably reproducible ^89^Zr-labeling protocol requiring a 15–30 min effort that involved simple and cost-effective quality control. The incorporation efficacy of the prelabeled agent was proven by the successful efficient labeling of liposomes and of human iPSCs. Due to the poor water solubility of oxine and oxinates, the use of DMSO and ethanol as a cosolvent was required. Thus, the short-term DMSO tolerance of the cells [20] and the ethanol’s impact on liposome size and stability [21] must be considered. However, DMSO-induced cell toxicity effects at concentrations of around ≥ 1% only became apparent upon much longer incubation times [22]. ^89^Zr-Labeled liposomes were injected into healthy mice to evaluate their stability and basic pharmacokinetic characteristics by PET. The pharmacokinetic profile and the 24 h organ distribution matched the characteristics of a nanosized labeled compound that was slowly excreted renally.

## 5. Conclusions

Prolonged PET imaging is an ideal tool in the development of lipid-based nanocarriers for drug delivery and cell therapies. For this objective, a reliable, reproducible and simplified ^89^Zr-radiolabeling method was developed and tested successfully in model liposomes and hiPCSs alike. This method may be adapted to other lipid-based nanomedicines or nanotheranostics, and to other cell species of interest.

## Data Availability

The datasets and PET images used and/or analyzed during the current study are available from the corresponding author on reasonable request.

## Supplementary Materials

The following are available online at https://www.mdpi.com/article/10.3390/pharmaceutics13071097/s1, Table S1: Radiolabeling yields of [89Zr]Zr(oxinate)4 until the extraction (60 min) and in the following stability samplings (2 h to 24 h). Table S2: Radiolabeling yields of [89Zr]Zr(oxinate)4–liposome complex until extraction (18–24 h) and in the following stability samplings (+ 24 h). Table S3: Radiolabeling yields of cells at different times of incubation and different DMSO concentrations. Table S4: 24 h ex vivo biodistribution results of [89Zr]Zr(oxinate)4–liposome-injected animals and the 89Zr control group (I.D./g organ). Table S5: 24 h ex vivo biodistribution results of [89Zr]Zr(oxinate)4–liposome-injected animals, and the 89Zr control group (I.D./whole organ). Figure S1: 3D-fused MicroPET/CT scans of [89Zr]Zr(oxinate)4–liposome-injected mice 24 h post-injection. Figure S2: Representative PET slices of the 89Zr control group (left), and the [89Zr]Zr(oxinate)4–liposome-complex-injected group (right) 24 h after IV injection. Figure S3: Particle size distribution of TargoSphere®.
