# Supplementary material for: Simplified 89Zr-Labeling Protocol of Oxine (8-Hydroxyquinoline) Enabling Prolonged Tracking of Liposome-Based Nanomedicines and Cells
Source: Pharmaceutics. 2021 Jul 18;13(7):1097. doi: 10.3390/pharmaceutics13071097 (PMC8309181; doi:10.3390/pharmaceutics13071097)
Supplement: Supplementary file 1 [file pharmaceutics-13-01097-s001.zip › pharmaceutics-1277366-supplementary.pdf]

pharmaceutics

MDPI

Supplementary Materials: Simplified ^{89}Zr-Labeling Protocol of Oxine (8-Hydroxyquinoline) Enabling Prolonged Tracking of Liposome-Based Nanomedicines and Cells

Andras Polyak, Jens P. Bankstahl, Karen F. W. Besecke, Constantin Hozsa, Wiebke Triebert, Rajeswara Rao Pannem, Felix Manstein, Thomas Borcholte, Marcus Furch, Robert Zweigerdt, Robert K. Gieseler, Frank M. Bengel and Tobias L. Ross

Table S1. Radiolabeling yields (%) of [^{89}Zr]Zr(oxinate)$_4$ until extraction (5 min to 60 min) and in the subsequentstability samplings (2 h to 24 h): Quick kinetics until a max. 98.7% labeling yield.

Entry	Before Extraction							Stability in PBS After Extraction		
	5 min	10 min	15 min	20 min	30 min	60 min	2 h	4 h	8 h	24 h
#1	22.4	73.5	94.2	95.9	98.7	97.5	97.1	94.9	95.6	93.7
#2	45.1	61.3	84.5	91.2	94.8	94.2	91.8	92.9	92.0	88.0
#3	27.8	73.5	86.0	93.1	97.1	91.3	-	-	-	-
#4	-	-	87.6	91.5	96.3	-	-	-	-	-
AVG ± SD	31.8 ± 11.9	69.4 ± 7.0	88.1 ± 4.3	92.9 ± 2.2	96.7 ± 1.6	94.3 ± 3.1	94.5 ± 3.7	93.9 ± 1.4	93.8 ± 2.5	90.9 ± 4.0

Table S2. Radiolabeling yields (%) of [^{89}Zr]Zr(oxinate)$_4$ liposome complex until extraction (5 min to 24 h) and in the subsequent stability samplings (+ 24 h): Relatively slow oxine incorporation kinetics.

	Labeling									Stability
Entry	5 min	10 min	15 min	20 min	30 min	60 min	6 h	18 h	24 h	+ 24 h
#1	9.8	18.0	30.0	35.0	42.0	48.2	81.6	98.1	94.6	96.2
#2	12.3	26.3	32.2	38.9	51.6	51.0	80.3	99.0	94.0	95.8
#3	8.6	15.2	26.2	26.9	34.3	66.3	78.2	95.6	97.1	98.3
#4	15.9	21.0	41.7	46.3	48.9	64.5	90.2	99.6	98.1	98.6
AVG ± SD	11.7 ± 3.2	20.1 ± 4.7	32.5 ± 6.6	36.8 ± 8.1	44.2 ± 7.7	59.2 ± 9.2	82.6 ± 5.3	98.1 ± 1.8	96.0 ± 2.0	97.2 ± 1.4

Table S3. Radiolabeling yields (%) of hiPSCs at different times of incubation and different DMSO concentrations: Quick kinetics in neutral conditions, ~50% max. labeling yield upon 30 min incubation.

		1% DMSO	2% DMSO	3% DMSO
**5 min**	**#1**	16.5	19.5	23.9
	**#2**	24.7	22.6	21.4
	**AVG ± SD**	20.6 ± 5.8	21.1 ± 2.2	22.7 ± 1.8
**10 min**	**#1**	45.3	47.6	45.5
	**#2**	35.8	39.0	40.1
	**AVG ± SD**	40.6 ± 6.7	43.3 ± 6.1	42.8 ± 3.8
**15 min**	**#1**	53.7	55.2	53.9
	**#2**	48.1	51.3	51.1
	**AVG ± SD**	50.9 ± 4.0	53.3 ± 2.8	52.5 ± 2.0
**30 min**	**#1**	48.0	48.8	51.7
	**#2**	47.1	52.3	54.8
	**AVG ± SD**	47.6 ± 0.6	50.6 ± 2.5	53.3 ± 2.2
**60 min**	**#1**	48.1	52.0	52.8
	**#2**	42.6	46.5	46.9
	**AVG ± SD**	45.4 ± 3.9	49.3 ± 3.9	49.9 ± 4.2
**6 h**	**#1**	45.0	44.0	42.0
	**#2**	39.0	40.0	39.0
	**AVG ± SD**	42.0 ± 4.2	42.0 ± 2.8	40.5 ± 2.1
**24 h**	**#1**	41.0	42.0	41.0
	**#2**	35.0	33.0	37.0

**AVG ± SD**	38.0 ± 4.2	37.5 ± 6.4	39.0 ± 2.8

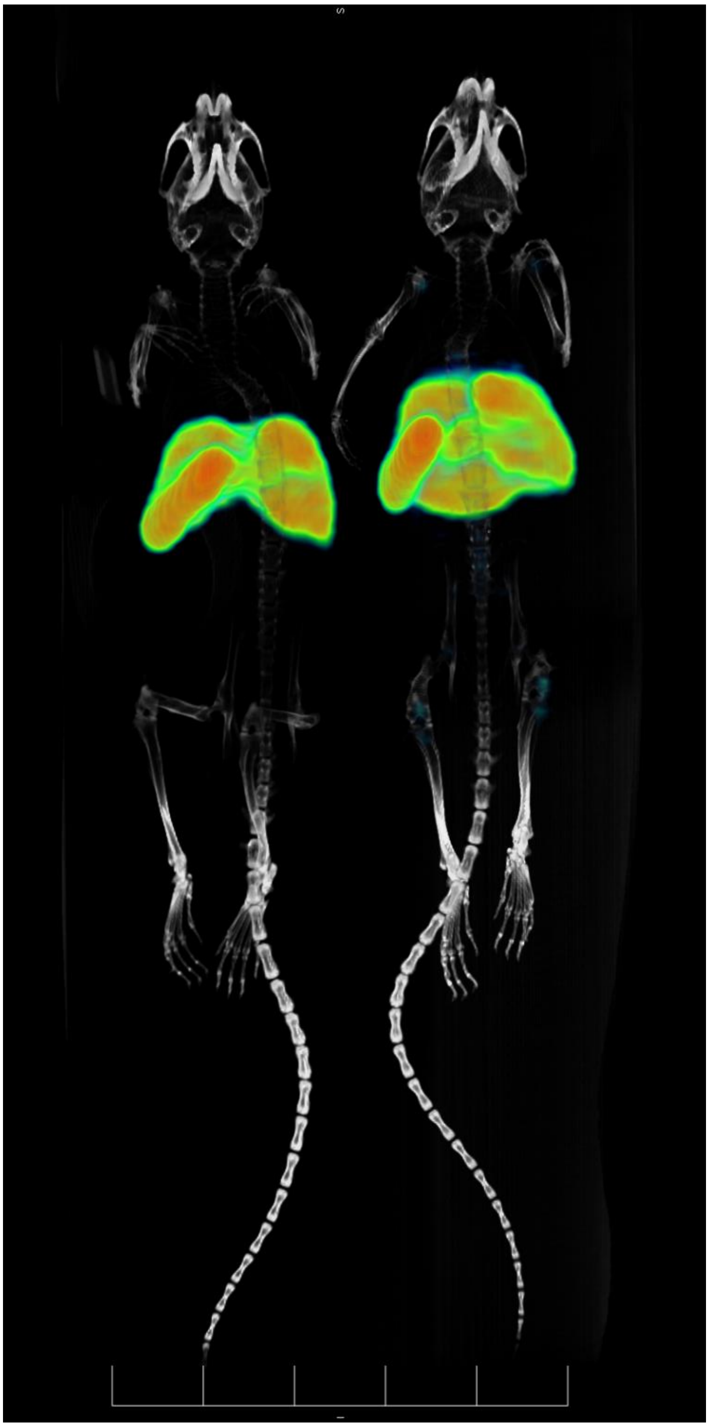

Figure S1. 3D-fused MicroPET/CT scans of [^{89}Zr]Zr(oxinate)$_4$–liposome-injected mice at 24 h post injection. High liver and spleen uptake, RES biodistribution. (Inveon DPET and CT120 small animal microPET/CT system; Siemens Healthineers, Erlangen, Germany.).

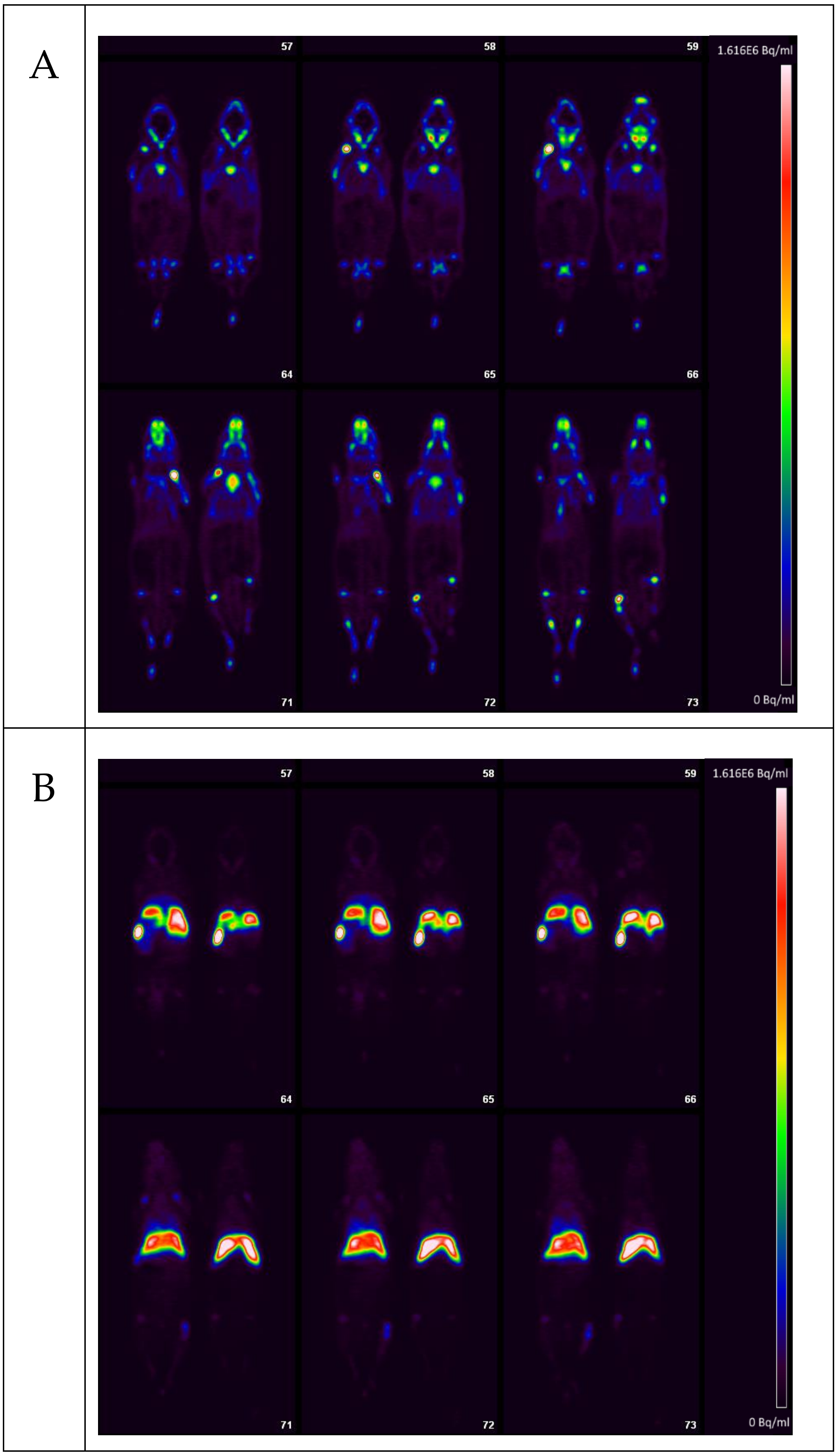

Figure S2. Representative PET slices of the ^{89}Zr control group (A), and the [^{89}Zr]Zr(oxinate)$_4$–liposome-complex-injected group (B) 24 h after IV injection. High liver and spleen uptake vs. bone uptake in the control group. (Inveon DPET and CT120 small animal microPET/CT system; Siemens Healthineers, Erlangen, Germany.).

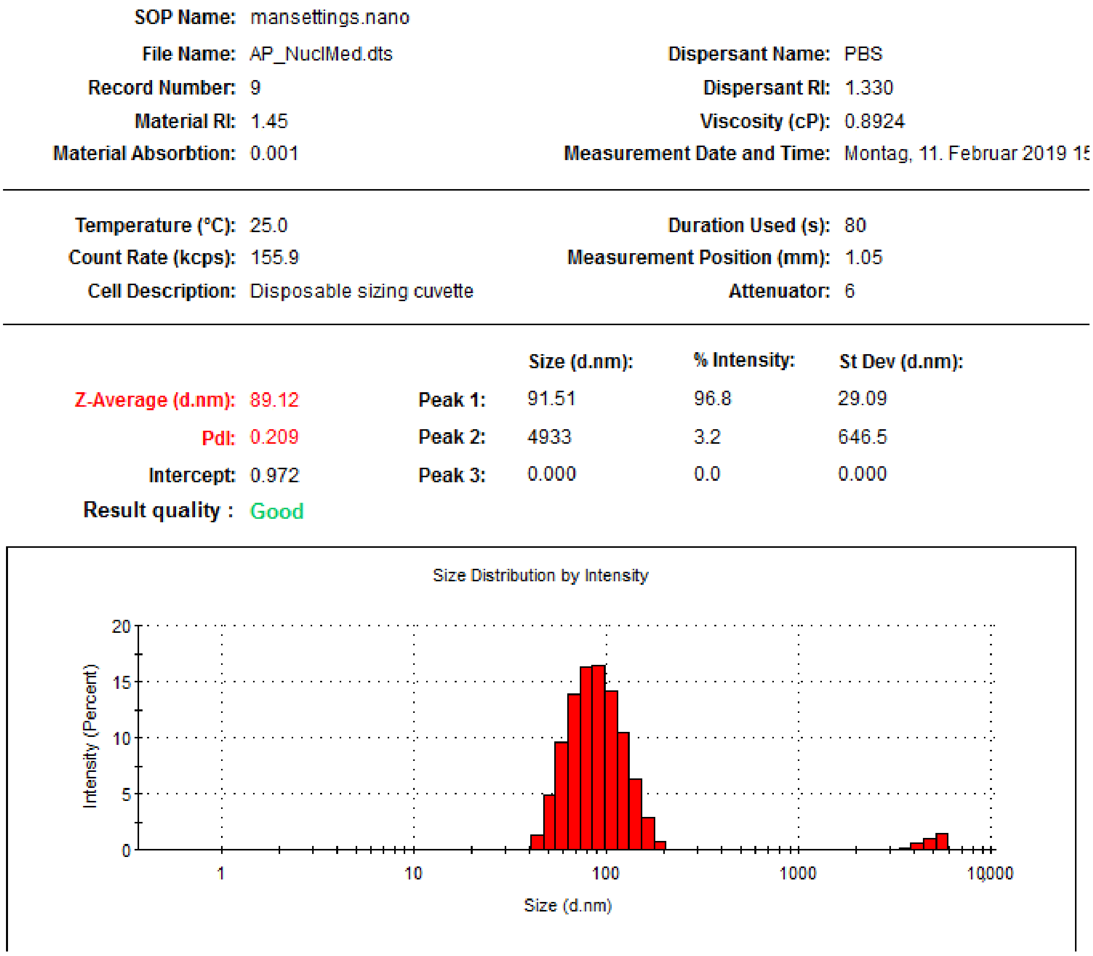

Figure S3. Particle size distribution of TargoSphere® [by Malvern Zetasizer Nano ZS dynamic light scattering (DLS) device.] (Translation: "Montag" = "Monday").

Table S4. 24 h ex vivo biodistribution results of [^{89}Zr]Zr(oxinate)$_4$–liposome-injected animals, and the ^{89}Zr control group (I.D./g organ percentages): High liver and spleen uptake vs. bone uptake in the control group.

	I.D./g organ %								
	[^{89}Zr]Zr(oxinate)$_4$–Liposome						^{89}Zr Control		
**Animal No.**	1	2	3	4	5	**AVG ± SD**	6	7	**AVG ± SD**
**Liver**	29.31	23.68	30.07	26.67	25.62	**27.07 ± 2.64**	1.30	1.15	**1.22 ± 0.11**
**Spleen**	75.43	61.28	65.31	88.78	63.90	**70.94 ± 11.33**	1.42	1.39	**1.40 ± 0.02**
**Lung**	7.49	4.98	11.35	7.02	3.18	**6.80 ± 3.07**	1.33	1.38	**1.36 ± 0.03**
**Kidney**	27.81	5.67	4.30	4.55	1.54	**8.77 ± 10.75**	1.24	1.23	**1.24 ± 0.01**
**Lymph Node**	2.21	2.88	6.59	1.17	1.89	**2.95 ± 2.13**	1.48	1.92	**1.70 ± 0.32**
**Salivary Gland**	1.53	1.24	1.98	1.40	0.78	**1.39 ± 0.43**	1.30	1.23	**1.26 ± 0.04**
**Blood**	1.79	1.69	1.46	1.15	0.51	**1.32 ± 0.51**	1.44	1.57	**1.51 ± 0.09**
**Urine**	0.34	0.24	1.50	1.34	1.02	**0.89 ± 0.58**	0.68	0.58	**0.63 ± 0.07**

Table S5. 24 h ex vivo biodistribution results of [^{89}Zr]Zr(oxinate)$_4$–liposome-injected animals, and the ^{89}Zr control group (I.D./whole organ percentages): High liver and spleen uptake vs. bone uptake in the control group.

	I.D./whole organ %								
	[^{89}Zr]Zr(oxinate)$_4$–Liposome						^{89}Zr Control		
Animal No.	1*	2*	3**	4**	5**	**AVG ± SD**	6**	7**	**AVG ± SD**
Liver	34.06	27.51	33.86	28.91	25.98	**30.06 ± 3.70**	1.92	1.66	**1.79 ± 0.19**
Spleen	4.55	3.68	7.18	6.03	5.41	**5.37 ± 1.35**	0.11	0.10	**0.11 ± 0.01**
Lung	-	-	2.17	1.84	0.56	**1.52 ± 0.85**	0.28	0.22	**0.25 ± 0.04**

Kidney	9.68	1.97	1.80	1.93	0.60	**3.20 ± 3.67**	0.57	0.53	**0.55 ± 0.03**
Salivary Gland	-	-	0.16	0.06	0.07	**0.09 ± 0.05**	0.10	0.11	**0.10 ± 0.01**
Blood*	2.61	2.47	2.13	1.68	0.75	**1.93 ± 0.75**	0.65	1.34	**0.99 ± 0.49**

* Extrapolated from I.D./g organ values and from calculated organ weights (using empiric body weight percentage). ** Calculated by real whole-organ activities and whole-organ weights, except for the blood values.
